# Cases Illustrating the Difficulties in Diagnosis and Treatment of Aneurisms of the Lower Extremity

**Published:** 1877-06

**Authors:** John R. Roberts


Cases Illustrating the Difficulties in Diagnosis and Treatment of Aneurisms of the Lower Extremity.—*By John R. Roberts, M.D.*—Internal aneurisms are proverbially difficult of accurate diagnosis, and their treatment is likewise fraught with uncertainty and risk. Similar lesions of the extremities, it would seem, ought to present much more characteristic symptoms and be in a greater degree amenable to medical and surgical treatment. The numerous mistakes that have been reported by experienced observers, however, show that this is not the case, for, notwithstanding the comparatively superficial position of the arterial trunks in the extremities, aneurismal dilatations are very serious lessons for the surgeon to attack. In the *American Journal of the Medical Sciences* for 1874, Dr. Stephen Smith has given us an account of a large number of cases where it was impossible to make an absolute diagnosis for or against aneurism until the question was solved by the result of treatment, or by the investigation of the pathologist after death. Again, Dr. Erskine Mason, in the same journal for January, 1877, reports a case in which he ligated the femoral artery for a case of supposed popliteal aneurism, but which was subsequently determined to be a sarcomatous growth. In the *Philadelphia Medical Times* for March 21, 1877, I recorded an instance of spontaneous aneurism of one of the branches, of dorsalis pedis artery, where there was great doubt as to whether the tumor was aneurismal or a ganglion receiving impulse from the subjacent artery. Professor Gross, at whose clinic the patient applied, cut down upon the small tumor to establish its nature, and, finding it to be an aneurism as suspected, enlarged the wound and ligated the anterior tibial near the ankle. Secondary hemorrhage supervened, and the artery was tied a second time higher up; but the patient died some days later, apparently from pyæmia.

Several somewhat similar cases have occurred recently in the wards of the Pennsylvania Hospital, which are of interest on account of the obscurity of diagnosis and the various methods of treatment adopted.

The first case was that of a large popliteal tumor, occurring in a negro aged 26 years, which presented few of the characteristics of aneurism, and yet, from the result of treatment, must be considered as such. The patient four years previous to admission had observed a swelling in the right ham, after having, he says, received a strain. He had no marked trouble until about two months since, when he found the tumor increasing; and three weeks ago this compelled him to resign work. There was no history of syphilis. On admission the right knee was semi-flexed, and the popliteal space was occupied by a large mass extending more to the inner side of the limb; the length of the tumor was seven inches, and the girth of the limb over the patella about nineteen inches. There was no pulsation and no thrill in the tumor, which was the seat of pain, and ausculation over the inner side revealed no murmur. The ear at this examination was unfortunately not applied to the outside of the mass. The tibial arteries could not be felt at the ankle. Over the heart there was heard a systolic murmur, probably the result of an attack of rheumatism which he had six years previously. The diagnosis lay between aneurism, deep abscess, and some heterologous growth, and in order to clear up the case an aspirating trocar was inserted into the tumor.

A stream of blood, which rapidly coagulated, flowed from the tube, but not in jets; there was no pus mixed with the blood, and pressure on the femoral did not immediately check the flow, though compression continued for some time, did seem to do so. The inguinal glands were enlarged, and the saphenous vein was somewhat distended. The day after the tumor was thus tapped, ausculation revealed a distinct, harsh, systolic murmur, heard especially over the outer aspect of the tumor, though it was also perceptible on the inner side of the leg. When the superficial femoral artery was compressed for some time, the swelling in the ham became much less tense and diminished in size. This was further tested by applying the Esmarch bandage and cutting off the circulation for five minutes, when the tumor became very much softer and the girth decreased one-half inch. An attempt to determine the difference in temperature of the feet was not very satisfactory.

It was plain that there was a hemorrhagic tumor in the popliteal space, for the result of the tapping was conclusive on that point. The needle had either entered into a hemorrhagic cancerous growth or aneurism. Which was the true condition was hard to determine; without the aspirator needle even this much would not have been decided, for the murmur could have readily been caused by pressure from any solid growth compressing the popliteal artery. The question of treatment had to be mooted, and was equally unsatisfactory. If the growth were cancerous, early amputation was the safest course; if aneurism of sacculated variety, ligation or compression was indicated; but if it were a dissecting aneurism, amputation might be required primarily, or secondarily after some less heroic procedure had been attempted. At the consultation the propriety of controlling the circulation and making an incision down to the tumor was discussed, which would give more certain data; for if the trouble were aneurismal, suppuration of the sac requiring free incission was not unlikely to occur whatever operative procedure were undertaken, and should the swelling be cancerous the wound would not be contra-indicated.

Finally, Dr. R. J. Levis, under whose care the patient was, decided to ligate the femoral artery in Scarpa's triangle and await results, as this was a more conservative procedure than amputation. This was accordingly done with a carbolized catgut ligature, and the wound closed. The murmur of course immediately ceased, but no marked dimunition in the size of the tumor occurred. The swelling, however, became very soft and fluctuating, so that it was confidently thought that suppuration within the sac of the supposed aneurism had taken place; and it was decided to withdraw some of the fluid with the aspirator. By accident this was postponed and the tumor never punctured. Very gradually the tumor then began to diminish, and the patient, who had been in good condition and had had little or no pain in the knee after the ligation, regained the use of the limb, and was discharged two months after the operation with only slight swelling remaining.

The second case was in the ward of Dr. William Hunt, and was very different from the first, in that the diagnosis was easily made and the result unexpected.

A. man, aged 40 years, whose occupation compelled him to climb awning-posts a great deal, had received years ago a gunshot wound of the inner side of the right leg, near the middle, where a small cicatrix could be seen. His friends say he had a partially-stiff knee for years, though he seems to think that he felt no pain until a week before admission, and he only relinquished work three days ago. On account of the patient being absolutely deaf, it is difficult to obtain an accurate history of the disease.

Examination showed a tumor, the size of a small orange, situated posterior to the head of the right tibia, which was the seat of an expansive pulsation, a distinct thrill, and a loud systolic murmur. Comprehension of the femoral at the groin caused these signs to disappear. The girth of the left leg at the same point was eleven and a half inches, while that of the right was thirteen and a quarter: when the circulation was cut off by pressure, the measurement fell to twelve and a quarter inches. No cardiac murmur was detected, and there was no history of syphilis. The radial artery did not appear atheromatous, and the dorsal artery of the foot was thought to be pulsating.

This, then, was a typical case of aneurism, and seemed to be a sacculated one, connected with the lower portion of the popliteal or one of the tibial arteries. Treatment by compression was deemed eminently adapted, as forced flexion exerted no influence, because the aneurism was below the joint and opposite the head of the tibia. This method was preferred to ligation, partly because of the success attending a case of external iliac aneurism treated by complete compression by Dr. Levis in the hospital in 1871. In this instance, according to Dr. Levis' report, published in the *Philadelphia Medical Times*, about a year later, the patient was kept etherized for five hours and a half, during which time the vessel was completely controlled by the pad of a compressor placed three and a half inches above Poupart's ligament. In the case before us, then, Dr. Hunt determined to try compression, and the pad, placed near the apex of Scarpa's triangle, was retained in position for twelve hours. During the first five or six hours the current was almost if not entirely arrested, because at times no pulsation whatever could be perceived in the tumor, though occasionally it was feebly felt. During the last half of the time, however, pulsation was marked, but I, who had the immediate supervision of the treatment, was afraid to increase the pressure lest gangrene should supervene, and determined to maintain partial occlusion for the rest of the time. Partial flexion of the knee was also employed, and the patient kept well narcotized. Subsequently the pulsation seemed diminished, but, owing to the inflammation of the skin caused by the instrument, no further pressure was applied. Forced flexion was continued without any change taking place. Twenty days later the compressor was again applied to the femoral, and the man enjoined to keep it screwed up as much as he could bear, but to relax the pressure occasionally when the pain oculd not be endured. This was treatment for two days, when it was found that the thrill had disappeared and that the pulsation was so feeble that at times it could be no longer certainly felt. The bruit remained. The tourniquet was used for a day or two longer in the same manner, but omitted at night. Consolidation was evidently taking place, and everything looked propitious; but soon a cloud appeared on the surgical horizon. On the fifth day after the patient began to use the compressor to produce partial arrest of the arterial current, swelling of the leg and great pain in the ham occurred. All compression was discontinued, and the limb enveloped in anodyne lotions. About the same condition of things lasted for four weeks: the swelling decreased a little and the pain gradually diminished, while the tumor was the seat of such feeble pulsation that it is impossible to state whether any actually existed. There seemed every reason to suppose that suppuration was about to occur in the aneurism, which had become filled with clot; but fluctuation did not present itself. At the end of this period, blebs appeared on the surface of the leg, and there were developed rather sudden symptoms of incipient gangrene, beginning in the neighborhood of the ankle. Further conservation was useless and delay hazardous, and accordingly, after consultation, amputation of the thigh was done six inches above the knee joint. Dr. Longstreth, the pathologist of the hospital, found the posterior tibial region filled with over a quart of laminated and soft black clot, which had pushed aside and forced itself into and around the muscles. No structure could be identified as a vessel or aneurismal sac. The popliteal artery, as far as the middle of the space, appeared normal, and then passed into the mass of clots. The head of the tibia and the bone just below were somewhat roughened, and a portion of the periosteum was removed. No bullet was found imbedded in the tissues, and at the time of dissection the parts presented no evidences of suppuration.

This was evidently an ordinary sacculated aneurism, which at some period of the treatment ruptured and became diffused. The symptoms of the trouble were so characteristic and decisive when first examined, that it seems certain that the aneurism was not diffused at that time; but after partial consolidation of the tumor had occurred, the weakened walls gave way and allowed extravasation among the muscular structures of the calf. This probably took place at the time when the limb rather suddenly became swollen and painful, during the use by the patient himself of partial compression. A case very similar to this was reported at a recent meeting of the New York Pathological Society, where a diffuse aneurism of the anterior tibial artery was followed by gangrene, necessitating amputation, and in which the first diagnosis of the case was cellulitis of the leg. In the present instance, it is impossible to say why the sac ruptured after partial consolidation of the aneurismal contents and a diminution of the blood-current had been effected by pressure upon the femoral; and yet it would appear that such was the fact. The patient, after the amputation of the thigh, did well, and was discharged cured.

A third case of aneurism was admitted by Dr. Levis on March 12, 1877.

The patient, aged 58, while, at work, nine or ten months previously, observed a swelling in the right groin, which always pulsated, and which increased gradually until it was as large as an orange. According to Dr. Hand's notes, the man gave no specific history, and said that the trouble was noticed after a hard day's work. The aneurism extended from one inch above Poupart's ligament down to the apex of Scarpa's triangle, and from the pubic symphysis outward to the ilio-pubic eminence. Pulsation could be felt when the fingers of the examiner were seven and a half inches apart. The dilatation therefore involved the iliac and femoral arteries just as the vessel passes from the abdominal cavity upon the thigh. In order to avoid, if possible, the ligation of the external iliac artery, it was determined to introduce a quantity of clean horsehair into the sac to favor deposition of fibrin and the consolidation of the aneurism. This method of treating aneurism was adopted in a case of thoracic aneurism by Dr. Levis some years ago, and would possibly have been successful if the tumor had not been of the diffused variety instead of sacculated, as was supposed. A long, delicate trocar was thrust into this ilio-femoral aneurism, and four long pieces of horsehair introduced through it. The trocar was then withdrawn in order to leave the hair curled up in the sac. Unfortunately, however, two peices, being entangled in the trocar, were dragged out with it, leaving within two pieces which measured each about sixteen and a half inches. In the course of three or four days the pulsation seemed slightly diminished, as though there was beginning fibrinous deposit, but at the same time inflammation of the overlying skin, tending towards sphacelation, occurred, and the sloughing threatened to open the sac. The patient stated that a somewhat similar attack of cutaneous inflammation had occurred once before. The danger of rupture of the sac from sloughing and fatal hemorrhage was so great that it was determined to wait no longer for the horsehair to induce coagulation, but to ligate the external iliac artery. Accordingly, four days after the introduction of the hair, an incision was made, from the crest of the ilium to within an inch of the pubic symphysis and half an inch above the crural arch, and a carbolized gut ligature applied. The patient showed symptoms of peritonitis afterwards, and died on the eleventh day subsequent to ligation. No autopsy was obtained, but the sac was opened, and the hairs and a mass of soft black clot found therein.

These three cases are worthy of study as showing the variety of phases that may be assumed by apparently simple and uncomplicated cases of aneurism. In the second case, cure seemed almost accomplished, but the rupture of the sac necessitated amputation by inducing gangrene; the third case unexpectedly required ligation on the cardiac side of the tumor, on account of sloughing of the superficial structures; while patient number one, when amputation was deemed almost the only chance of recovery, was cured by a simple ligation, without any bad symptoms.—*Med. Times.*

